# 1-(6-Chloro-1,3-benzothia­zol-2-yl)-2-[1-(4-meth­oxy­phen­yl)ethyl­idene]hydrazine

**DOI:** 10.1107/S1600536812032606

**Published:** 2012-08-11

**Authors:** Hoong-Kun Fun, Ching Kheng Quah, B. K. Sarojini, B. J. Mohan, B. Narayana

**Affiliations:** a X-ray Crystallography Unit, School of Physics, Universiti Sains Malaysia, 11800 USM, Penang, Malaysia; b Department of Chemistry, P. A. College of Engineering, Nadupadavu, Mangalore 574 153, India; c Department of Chemistry, Mangalore University, Mangalagangotri 574 199, Mangalore, India

## Abstract

The asymmetric unit of the title compound, C_16_H_14_ClN_3_OS, contains two independent mol­ecules (*A* and *B*) linked into dimers *via* N—H⋯N hydrogen bonds. The 1,3-benzothia­zol-2-yl ring system and the benzene ring form dihedral angles of 17.08 (8) and 8.63 (7)° in mol­ecules *A* and *B*, respectively.

## Related literature

For general background to and the biological, physical and chemical activities of hydrazone derivatives, see: Rollas & Küçükgüzel (2007 ▶); Naseema *et al.* (2010 ▶); Fouda *et al.* (2007 ▶); Dutkiewicz *et al.* (2010 ▶); Ali *et al.* (2004 ▶); Zeb & Yousuf (2011 ▶). For related structures, see: Fun *et al.* (2012*a* ▶,*b* ▶). For the stability of the temperature controller used for the data collection, see: Cosier & Glazer (1986 ▶). 
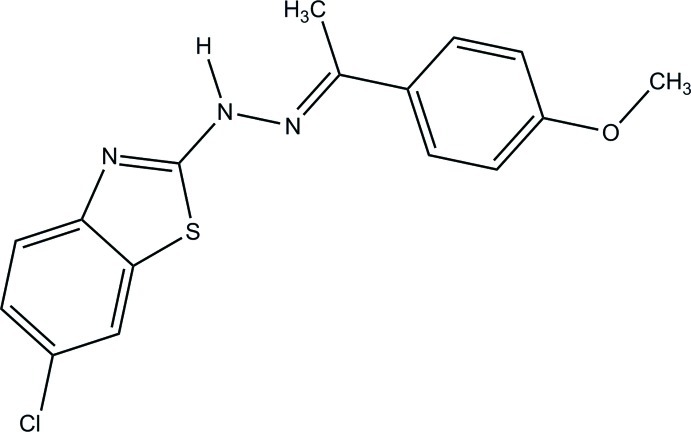


## Experimental

### 

#### Crystal data

C_16_H_14_ClN_3_OS
*M* *_r_* = 331.81
Triclinic, 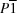

*a* = 8.5294 (1) Å
*b* = 9.3097 (1) Å
*c* = 19.8115 (3) Å
α = 87.999 (1)°
β = 78.091 (1)°
γ = 79.461 (1)°
*V* = 1513.32 (3) Å^3^
*Z* = 4
Mo *K*α radiation
μ = 0.40 mm^−1^
*T* = 100 K
0.25 × 0.20 × 0.06 mm

#### Data collection

Bruker SMART APEXII CCD area-detector diffractometer
Absorption correction: multi-scan (*SADABS*; Bruker, 2009 ▶) *T*_min_ = 0.908, *T*_max_ = 0.976
31608 measured reflections
6907 independent reflections
5393 reflections with *I* > 2σ(*I*)
*R*_int_ = 0.044

#### Refinement

*R*[*F* ^2^ > 2σ(*F* ^2^)] = 0.043
*wR*(*F* ^2^) = 0.088
*S* = 1.03
6907 reflections
409 parameters
H atoms treated by a mixture of independent and constrained refinement
Δρ_max_ = 0.36 e Å^−3^
Δρ_min_ = −0.40 e Å^−3^

### 

Data collection: *APEX2* (Bruker, 2009 ▶); cell refinement: *SAINT* (Bruker, 2009 ▶); data reduction: *SAINT*; program(s) used to solve structure: *SHELXTL* (Sheldrick, 2008 ▶); program(s) used to refine structure: *SHELXTL*; molecular graphics: *SHELXTL*; software used to prepare material for publication: *SHELXTL* and *PLATON* (Spek, 2009 ▶).

## Supplementary Material

Crystal structure: contains datablock(s) global, I. DOI: 10.1107/S1600536812032606/cv5320sup1.cif

Structure factors: contains datablock(s) I. DOI: 10.1107/S1600536812032606/cv5320Isup2.hkl

Supplementary material file. DOI: 10.1107/S1600536812032606/cv5320Isup3.cml

Additional supplementary materials:  crystallographic information; 3D view; checkCIF report

## Figures and Tables

**Table 1:** Hydrogen-bond geometry (Å, °)

*D*—H⋯*A*	*D*—H	H⋯*A*	*D*⋯*A*	*D*—H⋯*A*
N2*A*—H2N*A*⋯N1*B*	0.82 (2)	2.18 (2)	2.974 (2)	162 (2)
N2*B*—H2N*B*⋯N1*A*	0.88 (3)	2.13 (3)	2.983 (2)	166 (3)

**Fig. 1.**
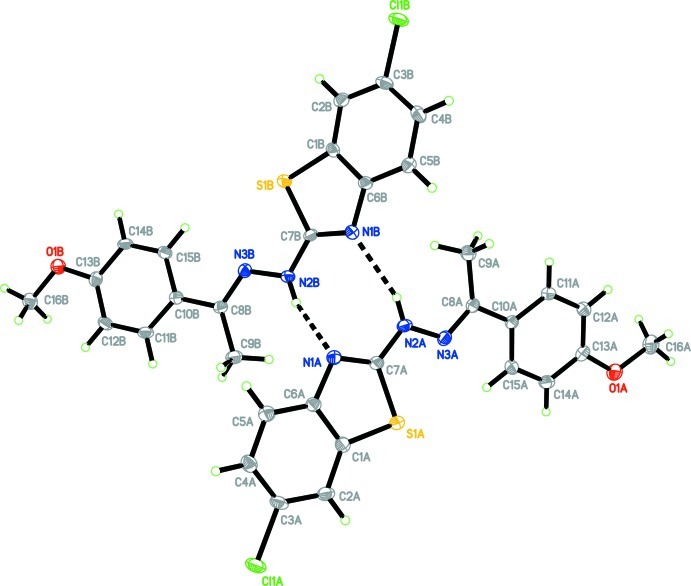
The content of asymmetric unit of (I) showing 50% probability displacement ellipsoids for non-H atoms. Intermolecular hydrogen bonds are shown as dashed lines.

## supplementary crystallographic information

### Comment

Hydrazones and their derivatives constitute a versatile class of compounds in organic chemistry. Recently, a lot of biologically important hydrazone derivatives with a number of functional groups have been synthesized. These compounds showed biological properties such as anticonvulsant, antidepressant, analgesic, antiinflammatory and antiplatelet activities (Rollas & Küçükgüzel, 2007). Hydrazone derivatives could be used in optical limiters and optical switches due to their optical limiting property (Naseema *et al.*, 2010). Hydrazone derivatives also act as corrosion inhibitors (Fouda *et al.*, 2007). Structures related to hydrazone derivatives have been reported (Dutkiewicz *et al.*, 2010; Ali *et al.*, 2004; Zeb & Yousuf, 2011). The present work describes the synthesis and crystal structure of the title compound, (I), prepared by the condensation of 1-(6-chloro1,3-benzothiazol-2-yl) hydrazine with 4-methoxyacetophenone in ethanol.

The asymmetric unit of (I) consists of two independent molecules, *A* and *B*, respectively (Fig. 1), with comparable geometries. In molecule *A*, the 1,3-benzothiazol-2-yl ring system (S1A/N1A/C1A–C7A, r.m.s. deviation = 0.029 Å) forms a dihedral angle of 17.08 (8)° with the benzene ring (C10A–C15A). The corresponding r.m.s. deviation and dihedral angle for molecule *B* are 0.010 Å and 8.63 (7)°, respectively. Bond lengths and angles are within normal ranges and are comparable with those observed in the related structures (Fun *et al.*, 2012*a*,*b*).

In the crystal structure, molecules *A* and *B* are interlinked *via* intermolecular N2A–H2NA···N1B and N2B–H2NB···N1A hydrogen bonds (Table 1) into dimers.

### Experimental

A mixture of 1-(6-chloro1,3-benzothiazol-2-yl)hydrazine (1.99 g, 10 mmol) and 4-methoxyacetophenone (1.5 g, 10 mmol) in ethanol (50 ml) was refluxed for 4 h. Completion of the reaction was monitored by TLC. After completion of the reaction, the reaction-mixture was poured into ice water. Brown colored solid separated out. The product obtained was washed with water and dried. The crude product was recrystallized from ethanol. Single crystals were grown by slow evaporation from solvent ethanol (m.p. 451–453 K).

### Refinement

N-bound H atoms were located in a difference Fourier map and isotropically refined with a restraint N—H = 0.85 (3) Å. The remaining H atoms were positioned geometrically and refined using a riding model with C—H = 0.95 or 0.98 Å and *U*_iso_(H) = 1.2 or 1.5*U*_eq_(C). A rotating group model was applied to the methyl groups.

### Crystal data

**Table:**

C_16_H_14_ClN_3_OS	*Z* = 4
*M**_r_* = 331.81	*F*(000) = 688
Triclinic, *P*1	*D*_x_ = 1.456 Mg m^−^^3^
Hall symbol: -P 1	Mo *K*α radiation, λ = 0.71073 Å
*a* = 8.5294 (1) Å	Cell parameters from 9939 reflections
*b* = 9.3097 (1) Å	θ = 2.2–32.9°
*c* = 19.8115 (3) Å	µ = 0.40 mm^−^^1^
α = 87.999 (1)°	*T* = 100 K
β = 78.091 (1)°	Plate, brown
γ = 79.461 (1)°	0.25 × 0.20 × 0.06 mm
*V* = 1513.32 (3) Å^3^	

### Data collection

**Table:**

Bruker SMART APEXII CCD area-detector diffractometer	6907 independent reflections
Radiation source: fine-focus sealed tube	5393 reflections with *I* > 2σ(*I*)
Graphite monochromator	*R*_int_ = 0.044
φ and ω scans	θ_max_ = 27.5°, θ_min_ = 2.1°
Absorption correction: multi-scan (*SADABS*; Bruker, 2009)	*h* = −11→11
*T*_min_ = 0.908, *T*_max_ = 0.976	*k* = −12→12
31608 measured reflections	*l* = −25→25

### Refinement

**Table:**

Refinement on *F*^2^	Primary atom site location: structure-invariant direct methods
Least-squares matrix: full	Secondary atom site location: difference Fourier map
*R*[*F*^2^ > 2σ(*F*^2^)] = 0.043	Hydrogen site location: inferred from neighbouring sites
*wR*(*F*^2^) = 0.088	H atoms treated by a mixture of independent and constrained refinement
*S* = 1.03	*w* = 1/[σ^2^(*F*_o_^2^) + (0.028*P*)^2^ + 1.121*P*] where *P* = (*F*_o_^2^ + 2*F*_c_^2^)/3
6907 reflections	(Δ/σ)_max_ = 0.001
409 parameters	Δρ_max_ = 0.36 e Å^−^^3^
0 restraints	Δρ_min_ = −0.40 e Å^−^^3^

### Special details

**Table:**

Experimental. The crystal was placed in the cold stream of an Oxford Cryosystems Cobra open-flow nitrogen cryostat (Cosier & Glazer, 1986) operating at 100.0 (1) K.
Geometry. All esds (except the esd in the dihedral angle between two l.s. planes) are estimated using the full covariance matrix. The cell esds are taken into account individually in the estimation of esds in distances, angles and torsion angles; correlations between esds in cell parameters are only used when they are defined by crystal symmetry. An approximate (isotropic) treatment of cell esds is used for estimating esds involving l.s. planes.
Refinement. Refinement of F^2^ against ALL reflections. The weighted R-factor wR and goodness of fit S are based on F^2^, conventional R-factors R are based on F, with F set to zero for negative F^2^. The threshold expression of F^2^ > 2sigma(F^2^) is used only for calculating R-factors(gt) etc. and is not relevant to the choice of reflections for refinement. R-factors based on F^2^ are statistically about twice as large as those based on F, and R-factors based on ALL data will be even larger.

### Fractional atomic coordinates and isotropic or equivalent isotropic displacement parameters (Å^2^)

**Table:**

	*x*	*y*	*z*	*U*_iso_*/*U*_eq_	
S1A	0.35667 (6)	0.80375 (5)	0.42848 (3)	0.01905 (12)	
Cl1A	0.92646 (7)	1.02723 (6)	0.36901 (3)	0.02725 (13)	
O1A	−0.47274 (18)	0.77478 (16)	0.68927 (7)	0.0251 (3)	
N1A	0.4366 (2)	0.71715 (17)	0.29901 (8)	0.0170 (4)	
N2A	0.1864 (2)	0.66677 (19)	0.36002 (9)	0.0179 (4)	
N3A	0.0764 (2)	0.69008 (17)	0.42147 (8)	0.0176 (4)	
C1A	0.5390 (2)	0.8395 (2)	0.37855 (10)	0.0173 (4)	
C2A	0.6504 (3)	0.9150 (2)	0.39716 (11)	0.0200 (4)	
H2AA	0.6336	0.9545	0.4422	0.024*	
C3A	0.7863 (3)	0.9303 (2)	0.34768 (11)	0.0202 (4)	
C4A	0.8153 (3)	0.8714 (2)	0.28172 (11)	0.0204 (4)	
H4AA	0.9115	0.8819	0.2493	0.025*	
C5A	0.7029 (2)	0.7971 (2)	0.26351 (11)	0.0195 (4)	
H5AA	0.7218	0.7564	0.2186	0.023*	
C6A	0.5629 (2)	0.7828 (2)	0.31144 (10)	0.0163 (4)	
C7A	0.3244 (2)	0.7215 (2)	0.35521 (10)	0.0162 (4)	
C8A	−0.0543 (2)	0.6348 (2)	0.43082 (10)	0.0163 (4)	
C9A	−0.0953 (3)	0.5383 (2)	0.37990 (11)	0.0219 (5)	
H9AA	−0.0378	0.5578	0.3333	0.033*	
H9AB	−0.2130	0.5589	0.3818	0.033*	
H9AC	−0.0618	0.4356	0.3915	0.033*	
C10A	−0.1681 (2)	0.6701 (2)	0.49761 (10)	0.0156 (4)	
C11A	−0.2985 (2)	0.5978 (2)	0.52026 (10)	0.0184 (4)	
H11A	−0.3157	0.5247	0.4917	0.022*	
C12A	−0.4048 (3)	0.6298 (2)	0.58367 (11)	0.0203 (4)	
H12A	−0.4929	0.5790	0.5981	0.024*	
C13A	−0.3803 (3)	0.7363 (2)	0.62527 (10)	0.0196 (4)	
C14A	−0.2529 (3)	0.8123 (2)	0.60313 (11)	0.0204 (4)	
H14A	−0.2375	0.8866	0.6314	0.025*	
C15A	−0.1490 (3)	0.7800 (2)	0.54017 (10)	0.0188 (4)	
H15A	−0.0630	0.8331	0.5254	0.023*	
C16A	−0.6045 (3)	0.6997 (3)	0.71405 (12)	0.0301 (5)	
H16A	−0.6595	0.7348	0.7605	0.045*	
H16B	−0.5628	0.5946	0.7155	0.045*	
H16C	−0.6821	0.7182	0.6831	0.045*	
S1B	0.10878 (6)	0.65349 (5)	0.10267 (3)	0.01748 (12)	
Cl1B	−0.14593 (6)	0.14927 (6)	0.10362 (3)	0.02510 (13)	
O1B	0.34041 (18)	1.34488 (15)	−0.10641 (7)	0.0228 (3)	
N1B	0.1780 (2)	0.55835 (17)	0.22159 (8)	0.0166 (4)	
N2B	0.2536 (2)	0.78361 (17)	0.18524 (9)	0.0168 (4)	
N3B	0.2594 (2)	0.87969 (17)	0.13039 (8)	0.0160 (3)	
C1B	0.0585 (2)	0.4861 (2)	0.13352 (10)	0.0159 (4)	
C2B	−0.0169 (2)	0.3934 (2)	0.10299 (10)	0.0183 (4)	
H2BA	−0.0480	0.4159	0.0599	0.022*	
C3B	−0.0446 (2)	0.2665 (2)	0.13819 (11)	0.0190 (4)	
C4B	0.0026 (2)	0.2299 (2)	0.20063 (11)	0.0197 (4)	
H4BA	−0.0171	0.1411	0.2229	0.024*	
C5B	0.0785 (2)	0.3235 (2)	0.23024 (10)	0.0179 (4)	
H5BA	0.1117	0.2992	0.2728	0.022*	
C6B	0.1055 (2)	0.4534 (2)	0.19703 (10)	0.0156 (4)	
C7B	0.1876 (2)	0.6651 (2)	0.17736 (10)	0.0149 (4)	
C8B	0.3288 (2)	0.9907 (2)	0.13189 (10)	0.0148 (4)	
C9B	0.4053 (3)	1.0242 (2)	0.18983 (11)	0.0217 (5)	
H9BA	0.3489	0.9867	0.2332	0.032*	
H9BB	0.3967	1.1302	0.1935	0.032*	
H9BC	0.5204	0.9777	0.1807	0.032*	
C10B	0.3327 (2)	1.0871 (2)	0.07057 (10)	0.0151 (4)	
C11B	0.4141 (2)	1.2052 (2)	0.06300 (11)	0.0183 (4)	
H11B	0.4673	1.2255	0.0983	0.022*	
C12B	0.4197 (2)	1.2945 (2)	0.00492 (11)	0.0190 (4)	
H12B	0.4767	1.3740	0.0006	0.023*	
C13B	0.3413 (2)	1.2662 (2)	−0.04644 (10)	0.0178 (4)	
C14B	0.2581 (2)	1.1491 (2)	−0.03972 (10)	0.0176 (4)	
H14B	0.2036	1.1300	−0.0748	0.021*	
C15B	0.2549 (2)	1.0610 (2)	0.01764 (10)	0.0169 (4)	
H15B	0.1988	0.9809	0.0214	0.020*	
C16B	0.4122 (3)	1.4732 (2)	−0.11283 (11)	0.0237 (5)	
H16D	0.4099	1.5161	−0.1586	0.036*	
H16E	0.5253	1.4474	−0.1070	0.036*	
H16F	0.3507	1.5442	−0.0773	0.036*	
H2NA	0.174 (3)	0.622 (2)	0.3271 (12)	0.020 (6)*	
H2NB	0.317 (3)	0.776 (3)	0.2152 (13)	0.033 (7)*	

### Atomic displacement parameters (Å^2^)

**Table:**

	*U*^11^	*U*^22^	*U*^33^	*U*^12^	*U*^13^	*U*^23^
S1A	0.0203 (3)	0.0234 (2)	0.0151 (2)	−0.0077 (2)	−0.0040 (2)	−0.0011 (2)
Cl1A	0.0276 (3)	0.0281 (3)	0.0337 (3)	−0.0151 (2)	−0.0154 (3)	0.0043 (2)
O1A	0.0232 (9)	0.0330 (8)	0.0177 (8)	−0.0078 (7)	0.0016 (7)	−0.0031 (6)
N1A	0.0158 (9)	0.0215 (8)	0.0153 (8)	−0.0059 (7)	−0.0049 (7)	0.0014 (7)
N2A	0.0173 (9)	0.0245 (9)	0.0138 (9)	−0.0071 (7)	−0.0038 (7)	−0.0028 (7)
N3A	0.0175 (9)	0.0219 (8)	0.0137 (8)	−0.0039 (7)	−0.0039 (7)	0.0015 (7)
C1A	0.0163 (11)	0.0174 (9)	0.0190 (10)	−0.0033 (8)	−0.0053 (9)	0.0028 (8)
C2A	0.0241 (12)	0.0192 (9)	0.0200 (11)	−0.0059 (8)	−0.0106 (9)	0.0009 (8)
C3A	0.0194 (11)	0.0188 (9)	0.0275 (12)	−0.0077 (8)	−0.0136 (10)	0.0044 (8)
C4A	0.0159 (11)	0.0221 (10)	0.0239 (11)	−0.0046 (8)	−0.0049 (9)	0.0043 (8)
C5A	0.0197 (11)	0.0210 (10)	0.0190 (10)	−0.0044 (8)	−0.0061 (9)	0.0011 (8)
C6A	0.0164 (11)	0.0166 (9)	0.0174 (10)	−0.0035 (8)	−0.0066 (9)	0.0024 (8)
C7A	0.0179 (11)	0.0168 (9)	0.0155 (10)	−0.0047 (8)	−0.0062 (8)	0.0019 (8)
C8A	0.0179 (11)	0.0165 (9)	0.0152 (10)	−0.0033 (8)	−0.0054 (8)	0.0022 (8)
C9A	0.0205 (11)	0.0258 (11)	0.0195 (11)	−0.0085 (9)	−0.0001 (9)	−0.0029 (8)
C10A	0.0169 (10)	0.0163 (9)	0.0147 (10)	−0.0030 (8)	−0.0056 (8)	0.0021 (8)
C11A	0.0212 (11)	0.0187 (9)	0.0177 (10)	−0.0071 (8)	−0.0064 (9)	0.0000 (8)
C12A	0.0187 (11)	0.0236 (10)	0.0198 (11)	−0.0077 (8)	−0.0035 (9)	0.0016 (8)
C13A	0.0207 (11)	0.0229 (10)	0.0147 (10)	−0.0015 (8)	−0.0045 (9)	0.0011 (8)
C14A	0.0218 (12)	0.0217 (10)	0.0193 (11)	−0.0053 (8)	−0.0062 (9)	−0.0021 (8)
C15A	0.0179 (11)	0.0207 (10)	0.0189 (10)	−0.0068 (8)	−0.0037 (9)	0.0022 (8)
C16A	0.0261 (13)	0.0383 (13)	0.0229 (12)	−0.0079 (10)	0.0036 (10)	−0.0006 (10)
S1B	0.0189 (3)	0.0201 (2)	0.0167 (2)	−0.0083 (2)	−0.0070 (2)	0.00203 (19)
Cl1B	0.0233 (3)	0.0256 (3)	0.0293 (3)	−0.0125 (2)	−0.0035 (2)	−0.0085 (2)
O1B	0.0315 (9)	0.0205 (7)	0.0195 (8)	−0.0110 (6)	−0.0078 (7)	0.0056 (6)
N1B	0.0169 (9)	0.0182 (8)	0.0156 (8)	−0.0066 (7)	−0.0023 (7)	−0.0001 (7)
N2B	0.0200 (9)	0.0190 (8)	0.0152 (9)	−0.0086 (7)	−0.0079 (8)	0.0020 (7)
N3B	0.0162 (9)	0.0179 (8)	0.0143 (8)	−0.0035 (7)	−0.0038 (7)	0.0015 (6)
C1B	0.0130 (10)	0.0180 (9)	0.0166 (10)	−0.0049 (8)	−0.0007 (8)	−0.0006 (8)
C2B	0.0143 (10)	0.0251 (10)	0.0158 (10)	−0.0045 (8)	−0.0027 (8)	−0.0028 (8)
C3B	0.0143 (10)	0.0200 (10)	0.0234 (11)	−0.0063 (8)	−0.0011 (9)	−0.0074 (8)
C4B	0.0182 (11)	0.0164 (9)	0.0231 (11)	−0.0056 (8)	0.0015 (9)	−0.0011 (8)
C5B	0.0189 (11)	0.0198 (10)	0.0149 (10)	−0.0039 (8)	−0.0026 (9)	−0.0009 (8)
C6B	0.0125 (10)	0.0192 (9)	0.0154 (10)	−0.0047 (8)	−0.0015 (8)	−0.0027 (8)
C7B	0.0141 (10)	0.0193 (9)	0.0117 (9)	−0.0037 (8)	−0.0030 (8)	−0.0026 (8)
C8B	0.0129 (10)	0.0163 (9)	0.0153 (10)	−0.0017 (7)	−0.0032 (8)	−0.0033 (7)
C9B	0.0287 (12)	0.0201 (10)	0.0202 (11)	−0.0081 (9)	−0.0110 (10)	0.0009 (8)
C10B	0.0128 (10)	0.0156 (9)	0.0162 (10)	−0.0010 (7)	−0.0026 (8)	−0.0016 (7)
C11B	0.0191 (11)	0.0204 (10)	0.0188 (10)	−0.0064 (8)	−0.0090 (9)	−0.0011 (8)
C12B	0.0200 (11)	0.0171 (9)	0.0224 (11)	−0.0082 (8)	−0.0058 (9)	0.0009 (8)
C13B	0.0196 (11)	0.0164 (9)	0.0162 (10)	−0.0018 (8)	−0.0022 (9)	0.0008 (8)
C14B	0.0172 (11)	0.0214 (10)	0.0165 (10)	−0.0054 (8)	−0.0065 (9)	−0.0037 (8)
C15B	0.0161 (10)	0.0166 (9)	0.0191 (10)	−0.0052 (8)	−0.0035 (8)	−0.0025 (8)
C16B	0.0276 (12)	0.0216 (10)	0.0234 (11)	−0.0084 (9)	−0.0063 (10)	0.0063 (9)

### Geometric parameters (Å, º)

**Table:**

S1A—C1A	1.743 (2)	S1B—C1B	1.748 (2)
S1A—C7A	1.757 (2)	S1B—C7B	1.7610 (19)
Cl1A—C3A	1.747 (2)	Cl1B—C3B	1.746 (2)
O1A—C13A	1.369 (2)	O1B—C13B	1.375 (2)
O1A—C16A	1.424 (3)	O1B—C16B	1.430 (2)
N1A—C7A	1.306 (3)	N1B—C7B	1.305 (2)
N1A—C6A	1.399 (2)	N1B—C6B	1.397 (2)
N2A—C7A	1.351 (3)	N2B—C7B	1.354 (2)
N2A—N3A	1.372 (2)	N2B—N3B	1.382 (2)
N2A—H2NA	0.82 (2)	N2B—H2NB	0.88 (2)
N3A—C8A	1.288 (3)	N3B—C8B	1.286 (2)
C1A—C2A	1.393 (3)	C1B—C2B	1.387 (3)
C1A—C6A	1.409 (3)	C1B—C6B	1.405 (3)
C2A—C3A	1.380 (3)	C2B—C3B	1.383 (3)
C2A—H2AA	0.9500	C2B—H2BA	0.9500
C3A—C4A	1.392 (3)	C3B—C4B	1.391 (3)
C4A—C5A	1.389 (3)	C4B—C5B	1.386 (3)
C4A—H4AA	0.9500	C4B—H4BA	0.9500
C5A—C6A	1.388 (3)	C5B—C6B	1.390 (3)
C5A—H5AA	0.9500	C5B—H5BA	0.9500
C8A—C10A	1.478 (3)	C8B—C10B	1.483 (3)
C8A—C9A	1.507 (3)	C8B—C9B	1.500 (3)
C9A—H9AA	0.9800	C9B—H9BA	0.9800
C9A—H9AB	0.9800	C9B—H9BB	0.9800
C9A—H9AC	0.9800	C9B—H9BC	0.9800
C10A—C11A	1.392 (3)	C10B—C11B	1.392 (3)
C10A—C15A	1.401 (3)	C10B—C15B	1.401 (3)
C11A—C12A	1.395 (3)	C11B—C12B	1.394 (3)
C11A—H11A	0.9500	C11B—H11B	0.9500
C12A—C13A	1.381 (3)	C12B—C13B	1.384 (3)
C12A—H12A	0.9500	C12B—H12B	0.9500
C13A—C14A	1.391 (3)	C13B—C14B	1.394 (3)
C14A—C15A	1.380 (3)	C14B—C15B	1.377 (3)
C14A—H14A	0.9500	C14B—H14B	0.9500
C15A—H15A	0.9500	C15B—H15B	0.9500
C16A—H16A	0.9800	C16B—H16D	0.9800
C16A—H16B	0.9800	C16B—H16E	0.9800
C16A—H16C	0.9800	C16B—H16F	0.9800
			
C1A—S1A—C7A	87.78 (10)	C1B—S1B—C7B	87.71 (9)
C13A—O1A—C16A	117.04 (17)	C13B—O1B—C16B	117.25 (16)
C7A—N1A—C6A	109.44 (16)	C7B—N1B—C6B	109.43 (16)
C7A—N2A—N3A	116.12 (17)	C7B—N2B—N3B	114.82 (16)
C7A—N2A—H2NA	119.3 (16)	C7B—N2B—H2NB	116.7 (16)
N3A—N2A—H2NA	124.5 (16)	N3B—N2B—H2NB	124.5 (16)
C8A—N3A—N2A	119.40 (17)	C8B—N3B—N2B	118.85 (16)
C2A—C1A—C6A	121.20 (19)	C2B—C1B—C6B	121.98 (18)
C2A—C1A—S1A	128.43 (16)	C2B—C1B—S1B	127.89 (15)
C6A—C1A—S1A	110.36 (15)	C6B—C1B—S1B	110.13 (14)
C3A—C2A—C1A	117.46 (19)	C3B—C2B—C1B	116.85 (18)
C3A—C2A—H2AA	121.3	C3B—C2B—H2BA	121.6
C1A—C2A—H2AA	121.3	C1B—C2B—H2BA	121.6
C2A—C3A—C4A	122.45 (19)	C2B—C3B—C4B	122.57 (18)
C2A—C3A—Cl1A	118.53 (16)	C2B—C3B—Cl1B	118.70 (16)
C4A—C3A—Cl1A	119.02 (17)	C4B—C3B—Cl1B	118.72 (16)
C5A—C4A—C3A	119.6 (2)	C5B—C4B—C3B	119.80 (18)
C5A—C4A—H4AA	120.2	C5B—C4B—H4BA	120.1
C3A—C4A—H4AA	120.2	C3B—C4B—H4BA	120.1
C6A—C5A—C4A	119.44 (19)	C4B—C5B—C6B	119.26 (18)
C6A—C5A—H5AA	120.3	C4B—C5B—H5BA	120.4
C4A—C5A—H5AA	120.3	C6B—C5B—H5BA	120.4
C5A—C6A—N1A	125.37 (18)	C5B—C6B—N1B	125.19 (17)
C5A—C6A—C1A	119.75 (18)	C5B—C6B—C1B	119.52 (17)
N1A—C6A—C1A	114.88 (18)	N1B—C6B—C1B	115.29 (17)
N1A—C7A—N2A	123.88 (18)	N1B—C7B—N2B	124.37 (17)
N1A—C7A—S1A	117.51 (15)	N1B—C7B—S1B	117.43 (15)
N2A—C7A—S1A	118.62 (15)	N2B—C7B—S1B	118.20 (14)
N3A—C8A—C10A	115.50 (17)	N3B—C8B—C10B	115.59 (17)
N3A—C8A—C9A	124.95 (18)	N3B—C8B—C9B	124.27 (18)
C10A—C8A—C9A	119.54 (18)	C10B—C8B—C9B	120.13 (17)
C8A—C9A—H9AA	109.5	C8B—C9B—H9BA	109.5
C8A—C9A—H9AB	109.5	C8B—C9B—H9BB	109.5
H9AA—C9A—H9AB	109.5	H9BA—C9B—H9BB	109.5
C8A—C9A—H9AC	109.5	C8B—C9B—H9BC	109.5
H9AA—C9A—H9AC	109.5	H9BA—C9B—H9BC	109.5
H9AB—C9A—H9AC	109.5	H9BB—C9B—H9BC	109.5
C11A—C10A—C15A	117.53 (18)	C11B—C10B—C15B	117.61 (18)
C11A—C10A—C8A	121.86 (18)	C11B—C10B—C8B	121.75 (17)
C15A—C10A—C8A	120.60 (18)	C15B—C10B—C8B	120.64 (17)
C10A—C11A—C12A	121.87 (19)	C10B—C11B—C12B	121.71 (18)
C10A—C11A—H11A	119.1	C10B—C11B—H11B	119.1
C12A—C11A—H11A	119.1	C12B—C11B—H11B	119.1
C13A—C12A—C11A	119.2 (2)	C13B—C12B—C11B	119.36 (19)
C13A—C12A—H12A	120.4	C13B—C12B—H12B	120.3
C11A—C12A—H12A	120.4	C11B—C12B—H12B	120.3
O1A—C13A—C12A	124.9 (2)	O1B—C13B—C12B	124.65 (18)
O1A—C13A—C14A	115.11 (18)	O1B—C13B—C14B	115.44 (17)
C12A—C13A—C14A	120.01 (19)	C12B—C13B—C14B	119.91 (18)
C15A—C14A—C13A	120.20 (19)	C15B—C14B—C13B	120.10 (18)
C15A—C14A—H14A	119.9	C15B—C14B—H14B	119.9
C13A—C14A—H14A	119.9	C13B—C14B—H14B	119.9
C14A—C15A—C10A	121.12 (19)	C14B—C15B—C10B	121.31 (19)
C14A—C15A—H15A	119.4	C14B—C15B—H15B	119.3
C10A—C15A—H15A	119.4	C10B—C15B—H15B	119.3
O1A—C16A—H16A	109.5	O1B—C16B—H16D	109.5
O1A—C16A—H16B	109.5	O1B—C16B—H16E	109.5
H16A—C16A—H16B	109.5	H16D—C16B—H16E	109.5
O1A—C16A—H16C	109.5	O1B—C16B—H16F	109.5
H16A—C16A—H16C	109.5	H16D—C16B—H16F	109.5
H16B—C16A—H16C	109.5	H16E—C16B—H16F	109.5
			
C7A—N2A—N3A—C8A	−176.68 (17)	C7B—N2B—N3B—C8B	−176.02 (17)
C7A—S1A—C1A—C2A	176.79 (18)	C7B—S1B—C1B—C2B	179.72 (19)
C7A—S1A—C1A—C6A	−1.74 (14)	C7B—S1B—C1B—C6B	−0.13 (15)
C6A—C1A—C2A—C3A	−0.9 (3)	C6B—C1B—C2B—C3B	0.2 (3)
S1A—C1A—C2A—C3A	−179.33 (15)	S1B—C1B—C2B—C3B	−179.58 (15)
C1A—C2A—C3A—C4A	−1.1 (3)	C1B—C2B—C3B—C4B	−1.2 (3)
C1A—C2A—C3A—Cl1A	178.88 (14)	C1B—C2B—C3B—Cl1B	177.64 (15)
C2A—C3A—C4A—C5A	1.6 (3)	C2B—C3B—C4B—C5B	1.0 (3)
Cl1A—C3A—C4A—C5A	−178.44 (15)	Cl1B—C3B—C4B—C5B	−177.90 (15)
C3A—C4A—C5A—C6A	0.1 (3)	C3B—C4B—C5B—C6B	0.3 (3)
C4A—C5A—C6A—N1A	176.79 (17)	C4B—C5B—C6B—N1B	178.88 (18)
C4A—C5A—C6A—C1A	−2.1 (3)	C4B—C5B—C6B—C1B	−1.2 (3)
C7A—N1A—C6A—C5A	179.64 (18)	C7B—N1B—C6B—C5B	178.79 (19)
C7A—N1A—C6A—C1A	−1.4 (2)	C7B—N1B—C6B—C1B	−1.1 (2)
C2A—C1A—C6A—C5A	2.5 (3)	C2B—C1B—C6B—C5B	1.0 (3)
S1A—C1A—C6A—C5A	−178.81 (14)	S1B—C1B—C6B—C5B	−179.17 (15)
C2A—C1A—C6A—N1A	−176.44 (17)	C2B—C1B—C6B—N1B	−179.14 (18)
S1A—C1A—C6A—N1A	2.2 (2)	S1B—C1B—C6B—N1B	0.7 (2)
C6A—N1A—C7A—N2A	−179.93 (17)	C6B—N1B—C7B—N2B	−179.15 (18)
C6A—N1A—C7A—S1A	0.0 (2)	C6B—N1B—C7B—S1B	1.0 (2)
N3A—N2A—C7A—N1A	−175.47 (17)	N3B—N2B—C7B—N1B	176.61 (18)
N3A—N2A—C7A—S1A	4.6 (2)	N3B—N2B—C7B—S1B	−3.5 (2)
C1A—S1A—C7A—N1A	1.05 (15)	C1B—S1B—C7B—N1B	−0.52 (16)
C1A—S1A—C7A—N2A	−179.01 (16)	C1B—S1B—C7B—N2B	179.61 (16)
N2A—N3A—C8A—C10A	−177.94 (15)	N2B—N3B—C8B—C10B	178.92 (16)
N2A—N3A—C8A—C9A	2.8 (3)	N2B—N3B—C8B—C9B	0.0 (3)
N3A—C8A—C10A—C11A	−168.14 (17)	N3B—C8B—C10B—C11B	−175.29 (18)
C9A—C8A—C10A—C11A	11.1 (3)	C9B—C8B—C10B—C11B	3.6 (3)
N3A—C8A—C10A—C15A	12.7 (3)	N3B—C8B—C10B—C15B	4.1 (3)
C9A—C8A—C10A—C15A	−168.03 (17)	C9B—C8B—C10B—C15B	−176.92 (18)
C15A—C10A—C11A—C12A	−1.6 (3)	C15B—C10B—C11B—C12B	−0.4 (3)
C8A—C10A—C11A—C12A	179.18 (18)	C8B—C10B—C11B—C12B	179.09 (18)
C10A—C11A—C12A—C13A	0.1 (3)	C10B—C11B—C12B—C13B	0.5 (3)
C16A—O1A—C13A—C12A	−0.6 (3)	C16B—O1B—C13B—C12B	−6.4 (3)
C16A—O1A—C13A—C14A	−179.99 (17)	C16B—O1B—C13B—C14B	174.81 (17)
C11A—C12A—C13A—O1A	−178.03 (18)	C11B—C12B—C13B—O1B	−178.90 (18)
C11A—C12A—C13A—C14A	1.3 (3)	C11B—C12B—C13B—C14B	−0.1 (3)
O1A—C13A—C14A—C15A	178.31 (17)	O1B—C13B—C14B—C15B	178.42 (17)
C12A—C13A—C14A—C15A	−1.1 (3)	C12B—C13B—C14B—C15B	−0.5 (3)
C13A—C14A—C15A—C10A	−0.5 (3)	C13B—C14B—C15B—C10B	0.7 (3)
C11A—C10A—C15A—C14A	1.9 (3)	C11B—C10B—C15B—C14B	−0.2 (3)
C8A—C10A—C15A—C14A	−178.95 (17)	C8B—C10B—C15B—C14B	−179.70 (18)

### Hydrogen-bond geometry (Å, º)

**Table:**

*D*—H···*A*	*D*—H	H···*A*	*D*···*A*	*D*—H···*A*
N2*A*—H2*NA*···N1*B*	0.82 (2)	2.18 (2)	2.974 (2)	162 (2)
N2*B*—H2*NB*···N1*A*	0.88 (3)	2.13 (3)	2.983 (2)	166 (3)
